# Increased Severity and Spread of *Mycobacterium ulcerans*, Southeastern Australia

**DOI:** 10.3201/eid2401.171070

**Published:** 2018-01

**Authors:** Alex Y.C. Tai, Eugene Athan, N. Deborah Friedman, Andrew Hughes, Aaron Walton, Daniel P. O’Brien

**Affiliations:** Barwon Health, Geelong, Victoria, Australia

**Keywords:** Mycobacteria ulcerans, bacteria, Mycobacteria ulcerans disease, Buruli ulcer, severity, spread, severe disease, tuberculosis and other mycobacteria, zoonoses, Australia

## Abstract

Reported cases of *Mycobacterium ulcerans* disease (Buruli ulcer) have been increasing in southeastern Australia and spreading into new geographic areas. We analyzed 426 cases of *M. ulcerans* disease during January 1998–May 2017 in the established disease-endemic region of the Bellarine Peninsula and the emerging endemic region of the Mornington Peninsula. A total of 20.4% of cases patients had severe disease. Over time, there has been an increase in the number of cases managed per year and the proportion associated with severe disease. Risk factors associated with severe disease included age, time period (range of years of diagnosis), and location of lesions over a joint. We highlight the changing epidemiology and pathogenicity of *M. ulcerans* disease in Australia. Further research, including genomic studies of emergent strains with increased pathogenicity, is urgently needed to improve the understanding of this disease to facilitate implementation of effective public health measures to halt its spread.

*Mycobacterium ulcerans* causes a necrotizing disease of skin and soft tissue known as Bairnsdale or Daintree ulcer in Australia and Buruli ulcer worldwide. The pathogenesis of *M. ulcerans* is driven by production of mycolactone, a polyketide-derived macrolide that triggers apoptotic cell death (*1*). The clinical spectrum of *M. ulcerans* disease ranges from usually painless nodules or ulcers on the limbs, to more severe forms of the disease, including edematous lesions (*2*). More severe disease has major implications for patients in terms of increased illness and long-term deformities, more complicated and prolonged treatments, and increased treatment costs (*3*,*4*). The World Health Organization (WHO) classification system classifies *M. ulcerans* disease by severity: category 1 represents mild disease, and categories 2 and 3 represent more severe disease (*5*). The disease is classified as a WHO neglected tropical disease and has become a major public health issue in sub-Saharan Africa and Australia.

In the state of Victoria in Australia, *M. ulcerans* disease was first observed in the Bairnsdale District in the 1930s and is now established on the Bellarine Peninsula (*6*). In recent years, the epidemiology of *M. ulcerans* disease in southern Victoria has noticeably changed, with rapidly increasing numbers of human cases reported per year and expansion into new geographic areas, including the Mornington Peninsula, an adjacent area with previously few cases (*7*). The reasons for this expansion are unknown but might be related to changing climate, population expansion, human activities, or a complex zoonotic cycle involving possums (*8*,*9*).

Clinicians from Barwon Health, a tertiary hospital in Geelong, Victoria, Australia, which is adjacent to the Bellarine Peninsula, manage a large proportion of reported case-patients in Victoria (*10*), and have recently observed an increasing number of severe cases of *M. ulcerans* disease with devastating consequences for patients. If true, this increase might suggest emergence of more pathogenic strains of *M. ulcerans* among other factors putting humans at risk. Therefore, the purpose of this study was to describe the epidemiology and pathogenesis of severe *M. ulcerans* disease, assess risk factors for its development, and clarify the evolution of severe disease in this region. Our findings might facilitate development of effective public health interventions to reduce illness and the costs of this disease.

## Methods

### Case-Patient Identification

All patients with confirmed *M. ulcerans* disease managed at Barwon Health during January 1998–May 2017 were included in this study. A confirmed *M. ulcerans* case was defined as presence of a lesion clinically suggestive of *M. ulcerans* infection plus 1 of the following: culturing of *M. ulcerans* from a lesion, a positive PCR result for IS2404 (*11*) for swab or biopsy specimens from a lesion (performed at the Victorian Infectious Diseases Reference Laboratory, Melbourne, Victoria, Australia), or histopathologic findings for an excised lesion showing a necrotic ulcer and presence of acid-fast bacilli.

### Data Collection

We prospectively collected clinical and demographic data by using Epi Info version 6 (Centers for Disease Control and Prevention, Atlanta, GA, USA). These data were information regarding age of patient; duration of symptoms at diagnosis; patient sex; geographic location of the case; concurrent conditions, such as diabetes mellitus and immune suppression; and type, site, and WHO category of lesions. We determined lesion size by measuring the extent of induration associated with the lesion with a ruler.

### Definitions

**Figure 1:**
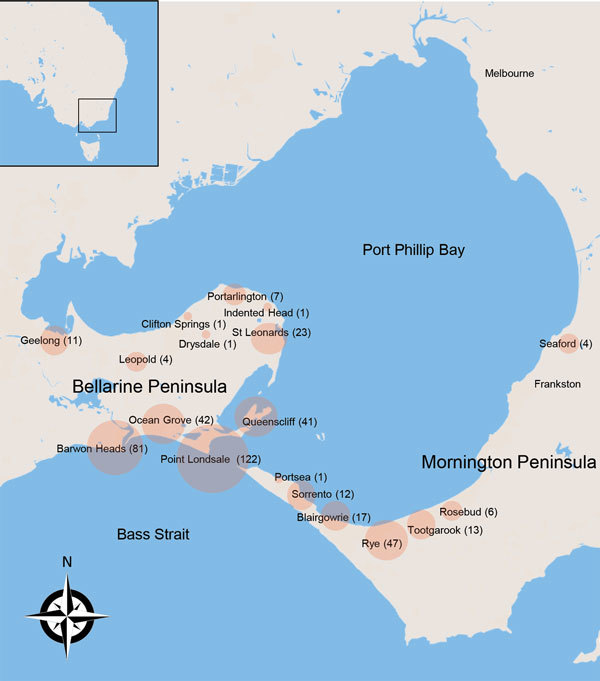
Locations (circles) of 426 cases of *Mycobacterium ulcerans* disease in Bellarine and Mornington Peninsulas, Barwon Health Cohort, Geelong, Victoria, Australia, January 1998–May 2017. Size of circles indicates number of cases, given in parentheses. Box in inset shows study region in southeastern Australia.

We defined severe disease as any lesion classified as WHO category 2 or 3 at diagnosis. Using WHO criteria, we defined category 1 lesions as single lesions <5 cm in diameter, category 2 lesions as single lesions 5–15 cm in diameter, and category 3 lesions as single lesions >15 cm in diameter; multiple lesions, lesions at a critical site (eye, breast, genitalia), or osteomyelitis (*5*). Plaque lesions were firm, painless, elevated lesions >3 cm in diameter with ill-defined edges. Edematous lesions were diffuse and usually nonpitting swelling with ill-defined margins involving part or all of a limb or other body part. A lesion over a joint was defined as a lesion overlying 1 of the following large joints: ankle, elbow, knee, wrist, or shoulder. Immune suppression was defined as current treatment with immunosuppressive medication (e.g., prednisolone) or an active malignancy. We classified cases by geographic location as acquired from either of the disease-endemic areas of the Bellarine or Mornington Peninsulas (Figure 1). Calendar years were categorized as time periods (1998–2004, 2005–2010, and 2011–2017) and included as a variable in analyses to assess whether time periods were associated with disease severity.

### Statistical Analysis

We analyzed data by using SPSS version 24 (IBM, Armonk, NY, USA). We compared categorical variables by using the Fisher exact test, χ^2^ test, or χ^2^ test for trend and categorical and numerical variables by using the Independent Student *t*-test, as appropriate between groups. A p value <0.05 indicated statistically significant differences.

We constructed a logistic regression model to assess the association of variables with severe disease. We obtained crude odds ratios (ORs) by performing univariate analysis and then performed multivariable analysis for age and sex a priori and all other variables showing an association with severe disease by univariate analysis (assessed by p<0.20): geographic location, position of lesion over a joint, time period (range of years of diagnosis), and diabetes mellitus. We determined p values for assessing the strength of the association of each variable with disease severity, which were controlled for all other variables in the multivariable model, by using the likelihood ratio test. In addition, we individually compared the association of severe disease at 5 body sites (ankle, elbow, hand, forearm, and knee) with severe disease at all other body sites combined, except for these 5 sites, by using univariate logistic regression.

### Ethics Approval

The study was approved by Barwon Health Human Research and Ethics Committee. All data were deidentified before analysis.

## Results

**Figure 2:**
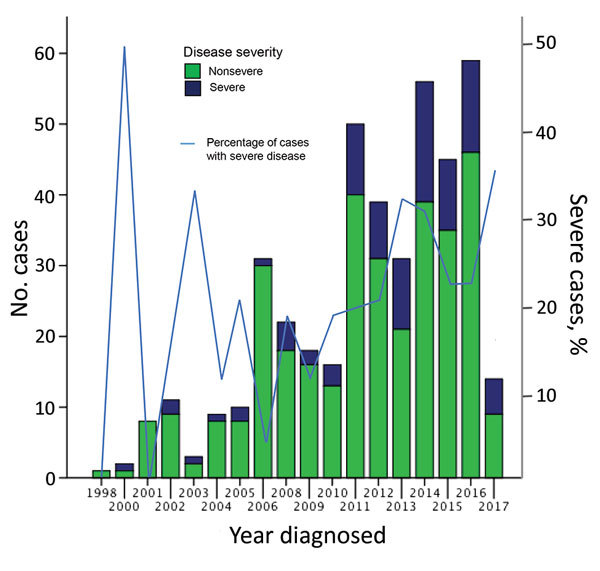
Proportion of severe and nonsevere cases of *Mycobacterium ulcerans* disease, Barwon Health Cohort, Geelong, Victoria, Australia, January 1998–May 2017.

A total of 446 case-patients with *M. ulcerans* disease were managed at Barwon Health during the study period. Twenty (4.5%) patients did not have WHO disease category recorded and were excluded. Thus, we included 426 case-patients in the study (Figure 1). We determined the number of cases included per calendar year stratified by disease severity (Figure 2).

### Baseline Characteristics

Median age for the 426 patients included in the analysis was 58 years (range 38–74 years), and 230 (54%) patients were male. Thirty-four (9.2%) cases were diagnosed during 1998–2014, 97 (24.2%) during 2005–2010, and 295 (66.6%) during 2011–2017. Median duration of symptoms before diagnosis was 42 days (interquartile range [IQR] 28–75 days).

We determined lesion type at diagnosis: 358 (84.0%) of lesions were ulcers, 27 (6.3%) were nodules, 36 (8.5%) were edematous lesions, and 4 (0.9%) were plaques. Diagnoses were made on the basis of a positive PCR result for 398 (93.4%) patients, positive histopathologic results for 23 (5.4%), and positive culture for 5 (1.2%). We determined that the WHO category of lesions was category 1 for 335 (78.6%) patients, category 2 for 46 (10.8%), and category 3 for 45 (10.6%), which resulted in 335 (78.6%) patients classified as having nonsevere disease and 91 (20.4%) as having severe disease. Lesions categorized as severe had an increased likelihood of being edematous (OR 19.52, 95% CI 8.83–43.17; p<0.001), and 21 (23.1%) of case-patients with severe disease had >1 lesion at presentation.

We determined sites of lesions and the proportion of severe disease per site (Table 1). When compared with all other lesions on the body combined apart from the ankle, elbow, forearm, hand, and knee, we found that there was a significantly higher likelihood of severe disease if lesions were located on the ankle (OR 3.99, 95% CI 2.12–7.50; p<0.001), elbow (OR 3.12, 95% CI 1.52–6.40; p = 0.002), or knee (OR 2.60, 95% CI 1.10–6.13; p = 0.029) (Table 1). We also identified additional baseline characteristics stratified by disease severity (Figure 2).

**Table 1:** Association between lesions at body sites and proportion of persons with severe *Mycobacterium ulcerans* disease, Barwon Health Cohort, Geelong, Victoria, Australia, January 1998–May 2017*

Site	Nonsevere disease, no. (%)	Severe disease, no. (%)	Crude odds ratio (95% CI)	p value
Ankle	40 (62.5)	26 (37.5)	3.99 (2.12–7.50)	<0.001
Elbow	32 (68.1)	15 (31.9)	3.12 (1.52–6.40)	0.002
Hand	16 (72.7)	6 (27.3)	2.49 (0.91–6.85)	0.077
Forearm	18 (75.0)	6 (25.0)	2.21 (0.82–6.01)	0.118
Knee	23 (71.9)	9 (28.1)	2.60 (1.10–6.13)	0.029
Arm	22 (88.0)	3 (12.0)	1	ND
Buttock	2 (66.7)	1 (33.3)	1	ND
Foot	17 (85.0)	3 (15.0)	1	ND
Leg	127 (86.4)	20 (13.6)	1	ND
Head	4 (80.0)	1 (20.0)	1	ND
Shoulder	3 (100.0)	0	1	ND
Thigh	18 (90.0)	2 (10.0)	1	ND
Trunk	4 (100.0)	0	1	ND
Wrist	9 (90.0)	1 (10.0)	1	ND

*ND, not determined.

### Patients with Severe Disease

Patients with severe disease were significantly older (median age 68 years, IQR 44–82 years) than patients without severe disease (median age 56 years, IQR 35–70 years; p<0.001). Univariate analysis showed that age (p = 0.004), geographic location (the Mornington Peninsula compared with the Bellarine Peninsula; p = 0.03), lesion located over a joint (p<0.001), time period (p = 0.01), and presence of diabetes mellitus (p = 0.06) were strongly associated with disease severity (Table 2). Crude analysis showed that sex, duration of symptoms before diagnosis, and immune suppression were not associated with disease severity (Table 2). Multivariate logistic regression analysis adjusting for age, sex, geographic location, position of lesion over a joint, time period, and diabetes mellitus showed that only the position of lesion over a joint (p<0.001), age (p = 0.006), and time period (p = 0.03) were significantly associated with severe disease (Table 2).

**Table 2:** Logistic regression analysis of adjusted and unadjusted associations between patient characteristics and severe *Mycobacterium ulcerans* disease, Barwon Health Cohort, Geelong, Victoria, Australia, January 1998–May 2017*

Characteristic	Nonsevere disease, no. (%)	Severe disease, no. (%)	Crude odds ratio (95% CI)	p value	Adjusted odds ratio (95% CI)	p value
Age, y						
≤15	31 (77.5)	9 (22.5)	1.62 (0.71–3.71)	0. 004	1.73 (0.73–4.12)	0.006
16–64	184 (84.8)	33 (15.2)	1		1	
≥65	120 (71.0)	49 (29.0)	2.28 (1.38–3.75)		2.34 (1.38–3.98)	
Sex						
F	156 (79.6)	40 (20.4)	1	0.66	1	0.51
M	179 (77.8)	51 (22.2)	1.11 (0.70–1.77		1.18 (0.72–1.94)	
Location						
Mornington Peninsula	72 (70.6)	30 (29.4)	1	0.03	1	0.24
Bellarine Peninsula	263 (81.2)	61 (18.8)	0.56 (0.33–0.93)		0.71 (0.40–1.26)	
Lesion over a joint						
No	228 (84.4)	42 (15.6)	1	<0.001	1	<0.001
Yes	107 (68.6)	49 (31.4)	2.67 (1.64–4.36)		2.71 (1.65–4.43)	
Time period						
1998–2004	29 (85.3)	5 (14.7)	1	0.01	1	0.03
2005–2010	85 (87.6)	12 (12.4)	0.82 (0.27–2.52)		0.88 (0.28–2.80)	
2011–2017	221 (74.9)	74 (25.1)	1.94 (0.73–5.20)		2.11 (0.74–5.99)	
Duration of symptoms before diagnosis, d						
≤75	241 (77.7)	69 (22.3)	1	0.76	NA	NA
>75	81 (81.0)	19 (19.0)	0.82 (0.46–1.44)			
Missing	13 (81.3)	3(18.8)	0.81 (0.22–2.91)			
Diabetes mellitus						
No	312 (79.8)	79 (20.2)	1	0.06	1	0.14
Yes	23 (65.7)	12 (34.3)	2.06 (0.98–4.32)		1.86 (0.82–4.23)	
Immune suppression						
No	310 (79.3)	81 (20.7)	1	0.31	NA	NA
Yes	25 (71.4)	10 (28.6)	1.50 (0.69–3.27)			

*Adjusted for age, sex, location, position of lesion over a joint, time period, and diabetes mellitus. NA, not applicable.

## Discussion

The findings of our study suggest a serious change in the epidemiology of *M. ulcerans* disease in southeastern Australia. There appears to be an increase in the proportion of severe cases in recent years, with a near doubling compared with earlier time periods to 25% of all case-patients who came to our health service during 2011–2017. More severe disease has major implications: case-patients with severe disease frequently require surgical treatment with tissue reconstruction (*2*) and hospitalization, and often have long-term deformities (*4*). Severe disease also causes major increases in cost of treatment (*3*). This finding is in contrast to nonsevere disease, which can usually be managed with oral antimicrobial drugs; outpatients have shown good outcomes (*12*–*14*). We also report a large proportion of severe cases that affected 1 of 5 patients in our cohort. However, these rates are lower than those reported for cohorts in Africa, in which the proportion of cases with severe disease (WHO categories 2 and 3 combined) was >70% (*15*–*17*). It has been postulated that strains in Africa are more virulent because of production of increased quantities and more potent forms of mycolactone (*18*).

The reason for the increasing proportion of severe cases in recent years is not clear but might be related to evolution of a more pathogenic strain of *M. ulcerans* in the region. In the area of *M. ulcerans* research, utility of whole-genome sequencing has been studied from an ecologic and epidemiologic perspective. Studies indicate a strong relationship between the genotype of an isolate and the geographic origin of disease at a national and regional level (*19*–*21*). However, there is no information available on the relationship between the genotype of an isolate and the clinical severity of disease in affected patients. In the related field of *M. tuberculosis* research, there has been increasing interest in using genomic information of individual disease isolates to predict clinical severity of disease in humans (phenotype), especially for *M. tuberculosis* strains with increased drug resistance (*22*). We advocate that genomic studies be conducted to explore whether particular *M. ulcerans* strains are associated with more severe disease, and if strains in disease-endemic regions are evolving over time to become more pathogenic. These studies might provide useful information for public health policy.

Other possible explanations for the increased proportion of severe cases of *M. ulcerans* disease could include environmental or climatic changes, which lead to a higher inoculum of organisms. In addition, there might have been a change in population dynamics or characteristics that make humans more susceptible to severe disease (*23*). Although human populations in disease-endemic regions are steadily increasing, these increases are not sufficient to explain the rapid increase in reported cases, and additional studies of these factors are needed.

Our analysis suggested that there was no major difference in the proportion of severe cases of *M. ulcerans* disease reported between the 2 peninsulas. This finding is consistent with our understanding of restricted genetic diversity of *M. ulcerans* from the same geographic location (*20*,*24*). A recent study of isolates from 11 disease-endemic countries in Africa identified only 2 specific *M. ulcerans* lineages; these lineages were subdivided into 4 major clusters, and most of these isolates were in cluster 1 (*25*). The spread of disease across both peninsulas in our study region, given their close geographic proximity, probably resulted from clonal expansion of the same genotype. This hypothesis is consistent with findings of a study of isolates from Australia in which 1 isolate from Frankston (Mornington Peninsula) had genomic sequences similar to those for isolates from Point Lonsdale and St. Leonards (Bellarine Peninsula) (*20*).

Apart from time period, we have identified 2 risk factors for severe *M. ulcerans* disease: a lesion situated over a joint and age. The reason for more severe disease occurring over joints is not clear. Reduced skin and subcutaneous tissue and the absence of muscle compared with adjacent regions might lead to lower tissue temperatures and increased growth of organisms (*26*,*27*). In addition, these factors might facilitate spread across tissue planes, and a reduced blood flow in the absence of muscle might relatively reduce local immune function. In contrast, increased tissue movement and physical stress over joints might increase lymph flow in the region and enhance spread of the infection, such as that hypothesized for hand, foot and mouth disease (*28*,*29*).

Our finding of older (>65 years) age as a risk factor for severe *M. ulcerans* disease is consistent with our previous studies that demonstrated that these patients were more likely to have multiple lesions or be categorized into WHO category 3 (*30*). This finding might be related to reduced immunity in patients in older age groups. Reduced immunity inhibits control of the mycobacterium and results in larger, more severe forms of *M. ulcerans* disease, which is similar to immunosuppressive effects of HIV (*31*). Reduced immunity in elderly persons might also be related to an increased prevalence of other concurrent conditions. Likewise, our study findings suggest that children might be at increased risk for severe disease, which might be related to relative immune suppression in an immune system that is still developing.

We found that, similar to findings from Africa (*32*), more prolonged signs and symptoms before diagnosis were not associated with severe disease. Therefore, severe disease is not directly related to a delay in diagnosis, as is often believed.

With the increasing number and severity of cases of *M. ulcerans* disease in Victoria, public health measures to prevent the disease are needed, although these measured are hampered because the environmental reservoir and mode of transmission of *M. ulcerans* are unknown. Current recommendations involve mosquito and insect bite preventive strategies, given some evidence that mosquitoes or another vector might be responsible for transmission (*33*,*34*). There is evidence of reduced risk for disease for persons outdoors in disease-endemic areas who reported regular use of insect repellent (*35*). In addition, sleeping under bed nets in Africa has been associated with a reduced incidence of disease (*36*). Possums have been postulated as a potential environmental reservoir in Australia (*8*,*9*), and more research is needed on the role of possums in the epidemiology of human *M. ulcerans* disease.

There were some limitations to this study. First, because we conducted an observational study, other unmeasured confounders associated with severe disease could affect the results. Second, there might be referral bias for case-patients managed at Barwon Health and increased numbers of severe cases referred over time because of the increasing expertise of clinicians in managing *M. ulcerans* disease. However, Barwon Health is the only tertiary referral center on the Bellarine Peninsula, and case-patients who are residents of this peninsula are seen by this health service regardless of disease severity. Therefore we believe that referral bias accounting for increasing disease severity over time is unlikely.

The epidemiology and pathogenicity of *M. ulcerans* disease in southeastern Australia is changing rapidly; we observed increases in numbers of cases per year and disease severity. Risk factors for severe disease include age, time period, and lesions located over a joint. Reasons for the changing epidemiology and pathogenicity are unknown but these factors urgently need to be identified and addressed to halt spread of this increasingly devastating disease.
